# Co-infection models of *Staphylococcus aureus* and respiratory viruses reveal synergistic interactions counteracted by a bithiazole derivative

**DOI:** 10.3389/fcimb.2026.1929910

**Published:** 2026-09-24

**Authors:** Giulia Radocchia, Marta De Angelis, Maria Grazia Martina, Francesca Barbieri, Rebecca De Gregorio, Sofia Venditti, Fabrizio Pantanella, Anna Teresa Palamara, Marco Radi, Serena Schippa, Lucia Nencioni

**Affiliations:** 1 Department of Public Health and Infectious Diseases, Sapienza University, Rome, Italy; 2 Department of Life Science, Health, and Health Professions, Link Campus University, Rome, Italy; 3 Department of Food and Drugs, University of Parma, Parma, Italy; 4 Department of Infectious Diseases, National Institute of Health, Rome, Italy

**Keywords:** antimicrobial activity, antivirals, bithiazole derivative, co-infections, coronavirus, influenza virus, Staphylococcus aureus

## Abstract

Viral-bacterial co-infections represent a major contributor to global morbidity and mortality. In influenza virus infections, secondary bacterial pneumonia occurs in approximately 20–30% of cases and is associated with an increased risk of shock, mechanical ventilation, and even mortality. Bacterial superinfections are frequent causes of mortality in influenza severe infections and have been a major cause of morbidity and mortality also in COVID-19 patients. *Staphylococcus aureus* has been identified as one of the most prevalent bacterial pathogens among hospitalized and non-hospitalized patients who have tested positive for SARS-CoV-2 or influenza virus infections. Generally, antibiotic therapy is administered to at-risk patients to preclude bacterial over-infections by eliminating susceptible pathogens, increasing selective pressure favoring antibiotic-resistant bacteria. Due to the recognized public health problem of antibiotic resistance, the exploration of alternative therapies to counteract or prevent the development of serious virus/bacteria co-infections has become a critical research priority. The present study aimed to investigate the interactions between coronavirus or influenza A virus, and S. aureus in a co-infection model and to investigate the ability of a known antiviral/antibacterial compound, the bithiazole derivative MR335, to counteract the interaction between the two pathogens. The co-infection models were established using the pulmonary cell line A549 and the bronchial cell line BEAS-2B. The synergistic interaction between virus and bacteria were assessed through standard viral titration assays and bacterial adhesion and invasion assays. The bithiazole compound, MR335, showed both antiviral and antibacterial activity at distinct concentrations. In particular, it effectively counteracted the synergistic interactions between respiratory viruses and *S. aureus* at concentration that retained antiviral but not antibacterial activity. Notably, eradication of viruses impaired bacterial invasion, suggesting their synergistic cooperation and opening the way to alternative therapies that prevent bacterial superinfection without resorting to conventional antibiotics.

## Introduction

Infections affecting the respiratory tract are a major global health problem due to the limited availability of truly effective drugs and the occurrence of drug resistance. The major complication of severe respiratory viral infections is the spread to the lower respiratory airways, often including secondary bacterial infection and resulting in severe pneumonia associated with an increased risk of shock, mechanical ventilation, and increased mortality (Kuiken and Taubenberger, 2008).

Much of the information on the severity of post-influenza bacterial pneumonia comes from retrospective studies conducted in the context of influenza pandemics. The first to be studied was the 1918-19 pandemic, known as the “Spanish flu”, in which most deaths were caused by bacterial pneumonia secondary to a viral respiratory tract infection. Studies conducted on lung tissue samples suggested that most of the estimated 20-60 million deaths were due to bacterial infections caused by *Streptococcus pneumoniae* or *Staphylococcus aureus* rather than the direct effects of the virus (Hoque et al., 2021). Later, in the pandemic of 1957-58, defined as the “Asian flu”, most deaths were attributed to pneumonia from secondary bacterial infection: 75% of confirmed fatal cases of influenza had bacteriological and histological evidence of bacterial pneumonia, mainly due to *S. aureus* or *S. pneumoniae*. In addition, in the most recent H1N1 pandemic of 2009, the well-known “Swine flu”, more than 50% of deceased had histological and microbiological evidence of bacterial pneumonia (Metzger and Sun, 2013). Indeed, early examinations of *postmortem* lung specimens from 77 fatal cases between May and August 2009 showed the presence of concomitant bacterial infection, caused by *S. pneumoniae* and *S. aureus* (Centers for Disease Control and Prevention (CDC), 2009). In patients with Coronavirus Disease 2019, co-infections have been identified as a primary cause of morbidity and mortality (Teklemariam et al., 2023). These co-infections may be characterized by the presence of bacteria, other respiratory viruses, or fungi (Chen et al., 2020; Fattorini et al., 2020; Rawson et al., 2020). However, the predominant causative agents of co-infections with the virus are bacteria (Vaillancourt and Jorth, 2020). A recent review of the literature indicates that co-infections in coronavirus disease were present in less than 4% of patients at the time of hospital admission. However, co-infections were frequent in patients requiring prolonged hospitalization (Westblade et al., 2021). As reported by Chen et al., approximately 50% of patients who died from the disease had a bacterial co-infection (Chen et al., 2020; Zhou et al., 2020). The most prevalent pathogens identified were *Staphylococcus aureus*, *Streptococcus pneumoniae*, and *Haemophilus influenzae*. However, in the context of hospitalized patients, *Pseudomonas aeruginosa*, *Klebsiella* spp., and *S. aureus* were identified as the most frequently isolated pathogens (Westblade et al., 2021). A similar outcome was observed in a study of 12.075 patients infected with SARS-CoV-2, which reported that the most prevalent bacterium associated with co-infection was *S. aureus* (Massey et al., 2020). *S. aureus* is a respiratory pathogen that can cause pneumonia and is also frequently reported as a co-infecting pathogen during influenza virus infections, with fatal consequences, especially if the strain involved in the co-infection is methicillin-resistant *S. aureus* (MRSA) (Mulcahy and McLoughlin, 2016). In a study of 221 adult patients with confirmed cases of severe acute respiratory syndrome (SARS) in Zhongnan Hospital in Wuhan, the incidence of co-infections was found to be significantly higher in severe cases than in non-severe cases (25.5% vs. 1.8%; *p*< 0.001) (Zhang et al., 2020). However, there is a paucity of data regarding the relationship between the seasonal coronavirus and *S. aureus* during co-infection.

It is known that SARS-CoV-2 has the capacity to compromise the structural integrity of the airway epithelium (Pizzorno et al., 2020). Nevertheless, the impact of SARS-CoV-2 on the pathogenic behavior of bacteria remains a subject that has not yet received sufficient attention. In the presence of a viral/bacterial co-infection, treatment is rendered more complicated and has the potential to worsen the prognosis and increase the likelihood of mortality (Chen et al., 2020; Zhou et al., 2020). To date, antibiotic therapy, with the objective of directly attacking bacteria, has been the most widely used treatment for the prevention and treatment of viral/bacterial co-infections. During the pandemic, a greater number of patients were treated with empirical antimicrobial therapy, thus promoting the global spread of drug-resistant bacteria (Chen et al., 2020, Barišić et al., 2025). Notably, the COVID-19 pandemic was associated with an approximately 15% increase in antimicrobial-resistant infections in the US (Sullivan et al., 2025). The necessity to explore alternative therapeutic interventions to antibiotics has become imperative in recent times, as the phenomenon of antibiotic resistance has emerged as a significant public health concern (Rawson et al., 2020).

Recently, the multi-dimensional pharmacological versatility of the bithiazole scaffold was investigated, revealing that opportune functionalization can lead to broad-spectrum antivirals that act on host targets rather than viral proteins, thereby reducing the likelihood of resistance selection (Tassini et al., 2017). Indeed, bithiazole derivatives inhibited not only members of the *Picornaviridae* family (e.g. enterovirus, poliovirus, rhinovirus), but also viruses belonging to the *Flaviviridae* (Zika) and *Coronaviridae* (SARS-CoV-2) families, targeting the host lipid kinase PI4KIIIβ (PI4KB) (Tassini et al., 2019; Martina et al., 2021, Martina et al., 2024). In addition, a second-generation of bithiazole derivatives has demonstrated the possibility of inhibiting the growth of Gram-positive and Gram-negative bacteria by targeting bacterial topoisomerase IV (Martina et al., 2022; Barbieri et al., 2024). Given this broad antimicrobial activity, the bithiazole scaffold is particularly attractive for investigating therapeutic strategies against infections involving both viral and bacterial pathogens. However, while previous studies have established that bithiazole derivatives can exert broad-spectrum antiviral activity through host-directed mechanisms and antibacterial activity through inhibition of bacterial topoisomerase IV, these properties have so far been investigated independently against specific viral or bacterial pathogens, rather than in models of co-infection.

Therefore, the objective of the present study was to establish a model of coinfection (influenza A virus/coronavirus and *S. aureus)* and to determine whether the antimicrobial properties of the bithiazole derivative (named MR335), already shown to exert antimicrobial activity against RNA viruses as well as Gram + bacteria (Martina et al., 2022, Martina et al., 2024), could interfere with the synergistic interaction between these pathogens, providing insights into its potential as a therapeutic approach against virus–bacteria coinfections.

## Materials and methods

### Cell culture and viruses’ production

Human lung adenocarcinoma cells (A549 cell line) were grown in DMEM medium supplemented with 10% fetal bovine serum (FBS), 0.3mg/ml glutamine, 100 U/ml penicillin, and 100 μg/ml streptomycin. Influenza virus A/Puerto Rico/8/34H1N1 (PR8/H1N1), influenza virus A/California/7/2009/H1N1 (pH1N1), influenza A/parrot/Ulster/73 (H7N1) or parainfluenza virus Sendai type 1 (SeV) were grown in allantoic cavities of 11-day-old embryonated chicken eggs. Viral suspension was inoculated in the allantoic cavity and incubated for 48 h at 37 °C, then infected eggs were maintained overnight at 4 °C. The allantoic fluid was collected and clarified by centrifugation (2500xg for 30 min). The recovered virus was used for the infection of A549 cells. Allantoic fluid from uninfected eggs was used as reference for mock infection.

Confluent monolayers of BEAS-2B were challenged with the Human Coronavirus (HCoV-229E) seasonal strain. After the viral adsorption, the cells were washed with phosphate-buffered saline (PBS) and then incubated with the medium supplemented with 2% FBS for 24 h. Virus production was determined in the supernatants of the infected cells by TCID_50_ (tissue culture infectious dose 50%) assay. Briefly, MRC-5 (Medical Research Council cell strain 5) cells were seeded in 96-well plates at a density of 2×10^4^ cells/well for 24 hours at 37 °C in an atmosphere of 5% CO_2_. Subsequently, cell monolayers were infected for 1 hour at 37 °C, using 10-fold dilutions of the samples, previously collected upon infection. After viral adsorption, cells were washed with PBS and 100 μL of fresh medium supplemented with 2% FBS will be added to each well for 24 hours at 37 °C. The number of wells showing positive CPE was then scored. TCID_50_/mL will be evaluated by means of the Reed and Muench method.

### Bacterial strains

A collection strain of *Staphylococcus aureus* ATCC 6538 was used in the study. The strain was stored at -80 °C in glycerol stocks. For experimental procedures, *S. aureus* was cultured directly from glycerol stock on Triptone Soy Agar plates (TSA, Oxoid, Basingstoke, UK) and incubated aerobically overnight at 37°C. A colony grown on TSA plate was inoculated in Brain Heart Infusion Broth (BHI, Oxoid, Basingstoke, UK) and incubated overnight at 37 °C with agitation (180 rpm). The concentration of the overnight bacterial suspension was then measured spectrophotometrically (BioPhotometer, Eppendorf, Hamburg, Germany).

### Compounds

The compound used was supplied in powder form, resuspended in 100% dimethyl sulfoxide (DMSO) (Sigma-Aldrich, St. Louis, MO, USA). Chemical structure, published data and reference to its synthesis are reported in **Table 1**.

**Table 1:** Chemical structure of MR335.

Compound	Reference paper to synthesis, characterization and biological evaluation
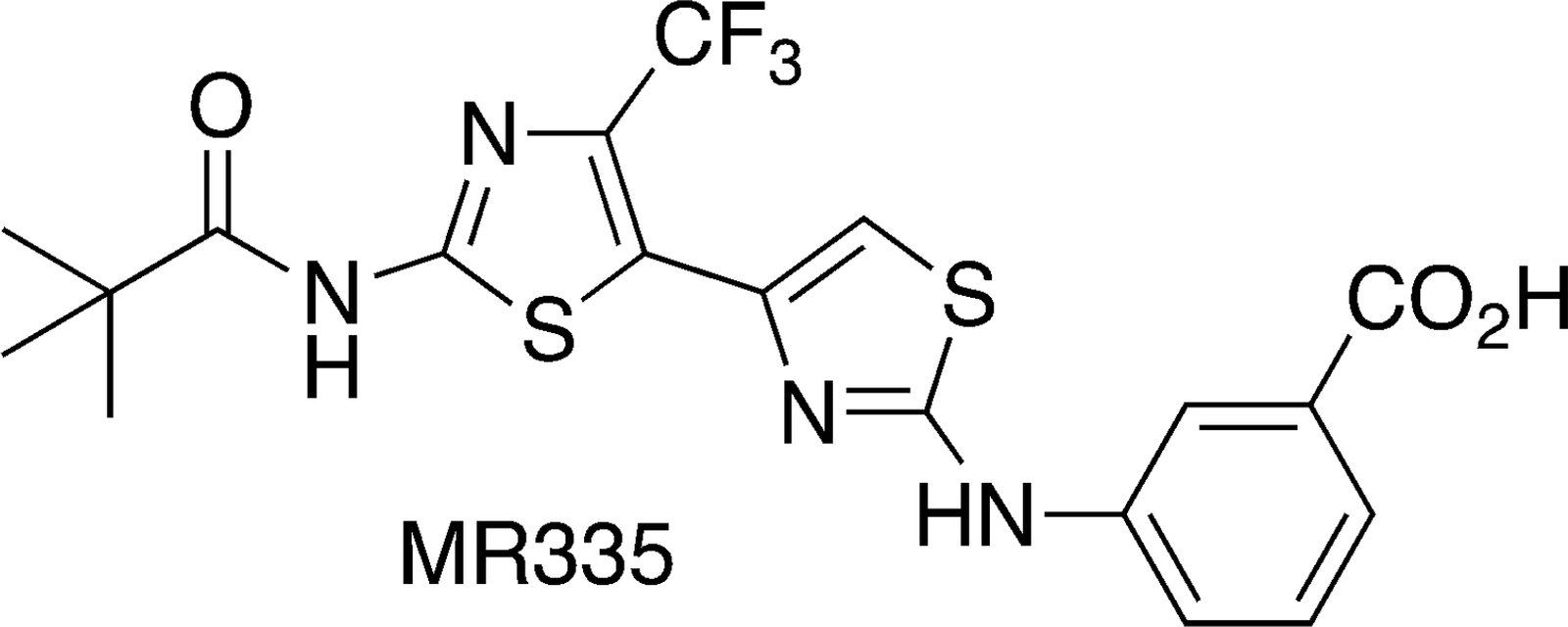	Martina, M. G.; Sannio, F.; Crespan, E.; Pavone, M.; Simoncini, A.; Barbieri, F.; Perini, C.; Pesce, E.; Maga, G.; Pedemonte, N.; Docquier, J-D.; Radi, M. *ChemMedChem* **2022**, *17*, e202200277.(compound **8b**) IC_50_ (PI4KB) = 7.1 ± 0.65 μM

### Cytotoxicity assay

The cytotoxicity of MR335 compound was assessed by using MTT (3-(4,5- dimethylthiazol-2-yl)-2,5-diphenyl tetrazolium bromide) reagent (Sigma-Aldrich, St. Louis, MO, USA) assay on A549 cell line. In a 96-well plate, 200 μl of cell suspension (10^4^ cells/well) were added to each well and the plates were incubated at 37 °C, 5% CO_2_. After 24 h, the culture medium was replaced with 200 μl of medium supplemented with MR335 compound at different concentrations (500, 400, 300, 200, 150, 100, 80, 60, 40, 20 μM). Untreated cells were used as control. After 24 h of contact, the culture medium was replaced with 200 μl of fresh medium added with 3-(4, 5-dimethylthiazol-2-yl)-2, 5-diphenyltetrazolium bromide (MTT) (5 mg/ml) (SERVA Electrophoresis GmbH, Heidelberg, Germany), then the plates were incubated for 4h at 37 °C, in a 5% CO_2_ atmosphere. Next, the culture medium with MTT was removed and 200 μl of dimethyl sulfoxide (DMSO) (Sigma-Aldrich, St. Louis, MO, USA) were added to each well to dissolve the formazan crystals formed in the live cells. After 15 minutes of incubation, absorbance was measured at 570 nm, with the reference set at 620 nm, using the automatic microplate reader, Tecan Sunrise (Kumar et al., 2018). For solvent control, the cell viability was compared with the DMSO-treated cells, at the same volumes used for MR335 dilutions.

### Co-infection models

Co-infection models between respiratory viruses and *S. aureus* were settled, referring to previous studies with some modifications (Oliva and Terrier, 2021; Sura et al., 2022; Goncheva and Heinrichs, 2023). In particular, co-infection models of *S. aureus* and PR8/H1N1 strain on A549 alveolar cell line and of *S. aureus* and HCoV-229E strain on BEAS-2B bronchial cell line were set up.

For this purpose, A549 or BEAS-2B cells were seeded in 24-well culture plates at a concentration of 1,5×10^5^ cells/mL, resuspended in complete DMEM medium and incubated at 37°C, 5% CO_2_. After 24 h of incubation, the cells were infected with PR8/H1N1 or HCoV-229E virus at MOI 0,1, in DMEM at 2% FBS, without antibiotics. After 22 h of viral infection, the cell monolayers were infected with *S. aureus* at a multiplicity of infection of 1 for A549 and 10 for BEAS cell line. The MOI was independently optimized for each cell line based on preliminary experiments aimed at identifying conditions yielding reproducible and quantifiable adhesion and invasion.

The cells were then incubated for 2 h at 37 °C, 5% CO_2_. At the end of the 2 h of infection, supernatants were collected for viral titration assays. In addition, bacterial adhesion and invasion were evaluated. Cells were infected with *S. aureus* alone or virus alone in order to compare the results with those of the co-infection models. In parallel, the same experimental conditions were used to evaluate the efficacy of MR335 compound in interfering with virus-bacteria synergistic interactions. To this aim, the compound was added after viral adsorption and maintained for the following 24 h post-infection.

### Antiviral activity of bithiazole compound

Confluent monolayers of A549 or BEAS-2B cells were challenged with influenza virus A/Puerto Rico/8/34 H1N1 (PR8) or HCoV-229E, respectively, for 1 h at 37 °C. Then, cells were washed with PBS and incubated or not with MR335 compound diluted in medium supplemented with 2% of FBS for 24 h post-infection. Mock infection was performed with the same dilution of allantoic fluid. Viral titration was performed by TCID_50_ or Hemagglutination assay (HAU) method.

### In-cell western assay

A549 cells were grown in 96-well plates (2 × 10^4^ cells/well) and incubated with the supernatants of infected and treated cells for 1 h at 37 °C. After 24 h of infection, cells were fixed with 4% paraformaldehyde (Santa Cruz Dallas, TX, USA), permeabilized with 0.1% Triton X-100, and incubated with Odyssey Blocking buffer (LI-COR Biosciences, Lincoln, NE, USA) for 1 h. Anti-Hemagglutinin (HA) or anti-influenza (Flu) antibodies (Santa Cruz Biotechnology, Santa Cruz, CA, USA) diluted in Odyssey Blocking Buffer were used to stain the cell monolayer at 4 °C overnight. Then, cells were washed and stained with fluorochrome-conjugated secondary antibodies together with Cell Tag (LI-COR Biosciences, Lincoln, NE, USA) (LI-COR Biosciences, Lincoln, NE, USA), a control for cell monolayer integrity, for 1 h at room temperature. The plates were analyzed by the Odyssey Imaging System (LI-COR, Lincoln, NE, USA). LI-COR Image Studio software was used to determine the integrated intensities of fluorescence, and the Relative Fluorescence Unit (RFU) was expressed as a percentage compared with untreated infected cells (100%).

### Real time PCR

For Hemagglutinin (HA) and coronavirus Nucleoprotein (N) relative quantification, total RNA was isolated from cell lysates (Total RNA Purification Plus Kit, Norgen Biotek, Thorold, ON, Canada) and used as a template for generating cDNA (iScript cDNA Synthesis Kit, Bio-Rad, Milan, Italy). An aliquot of the cDNA was subjected to 40 cycles of RT-qPCR amplification (95 °C, 10 sec; 60°C, 30 sec) using iQ SYBR Green Supermix and a LightCycler iQ 5 (Bio-Rad, Milan, Italy). Primer sequences are reported below:

Influenza HA (Hemagglutinin) forward 5’ATTCCGTCCATTCAATCCAGAGG 3’ and reverse 5’ ACCATCCATCTATCATTCCAGTCC 3’; HCoV-229E Nucleocapsid (N) forward 5’-AGGCGCAAGAATTCAGAACCAGAG-3’ and reverse 5’-AGCAGGACTCTGATTACGAGAAAG-3’.

### Evaluation of antibacterial activity of bithiazole compound

The antibacterial activity of the synthetic bithiazole compound was tested on the Gram-positive *S. aureus* strain. To evaluate the efficacy in growth inhibition, a bacterial suspension of *S. aureus* 1x10^8^ CFU/ml was seeded in a 96-well plate in 200 μl of BHI broth containing different concentrations of the compounds (500, 400, 300, 200, 150, 100, 50 and 25 μM). As a positive control, the bacterial suspension was seeded in the absence of any substance. Likewise, to assess the effect of DMSO on bacterial growth, quantities of DMSO equal to those in which the compound was resuspended at the different concentrations were placed in contact with the bacterial suspensions. After 24 h of incubation at 37 °C, the contents of each well were collected, diluted, and seeded on TSA plates for bacterial colony counts.

### Evaluation of antibiofilm activity of bithiazole compound

The antibacterial activity of MR335 compound was tested on the *S. aureus* ATCC 6538 biofilm. Bacterial biofilms were formed on a 96-well plate inoculated with 100 µl of a 1x10^8^ CFU/ml bacterial suspension and incubated 4 h at 37 °C. After the first 4 h of adhesion and microcolony formation, the supernatant was removed from each well and plates were rinsed with 100 µl of physiological saline (PS). Then, 100 µl of fresh medium were added and the plates were incubated at 37°C for an additional 20 h. The anti-biofilm activity of the substance was tested on both the 24 h formed biofilm and the forming biofilm. For the formed biofilm, after the 24 h incubation, the wells were rinsed with PS and 100 µl of BHI containing different concentrations of MR335 (500, 300 and 50 µM) were added.

For the inhibition of biofilm formation, to better understand at which stage of biofilm development the substances acted, the experiment was performed under 3 different conditions: (B) a 96-well plate was inoculated with 100 µl of a 1x10^8^ CFU/ml bacterial suspension resuspended in BHI broth with the addition of different concentrations of the compound (500, 300 and 50 µM). After 4 h of biofilm formation, the supernatant was removed from each well and the plate was rinsed with 100 µl of PS. Then 100 µl of fresh BHI medium were added to each well and the plate was incubated at 37 °C for 24 h; (BA) a 96-well plate was inoculated with 100 µl of a 1x10^8^ CFU/ml bacterial suspension in BHI broth with the addition of different concentrations of the compound (500, 300 and 50 µM). After 4 h of biofilm formation, the supernatant was removed from each well and the plate was rinsed with 100 µl of PS. Then 100 µl ml of fresh BHI medium containing the different concentrations of MR335 were added to the wells and the plate was incubated at 37 °C for 24 h; (A) a 96-well-plate was inoculated with 100 µl of a 1x10^8^ CFU/ml bacterial suspension in BHI broth and after 4 h of biofilm formation, the supernatant was removed from each well and the plate was rinsed with 100 µl of PS. Then 100 µl of BHI containing different concentrations MR335 were added, and the plate was incubated at 37 °C for 24 h.

For each condition, after 24 h of incubation with the compound, quantification of biofilm biomass was assessed using the crystal violet (CV) assay (Radocchia et al., 2025).

### *S. aureus* adhesion and invasion assay on airway cell lines

The adhesive and invasive ability of *S. aureus* was assessed through the adhesion and invasion assay. To evaluate adhesion, after 2 h of bacterial infection, the wells were washed three times with PBS and cells were detached and lysed with 500 μL 0.1% (v/v) Triton X-100 (Sigma-Aldrich, St. Louis, MO, USA) per well and incubated for 5 min at room temperature (rt). The well content was then diluted in sterile PBS and seeded on TSA plates for bacterial colony counts. The colonies were counted and normalized to the number of eukaryotic cells present in the well. To evaluate the invasion ability of *S. aureus*, after 2 h of bacterial infection, the cells were washed twice with PBS before the addition of DMEM containing 200 μg/mL of gentamicin (CN) (Sigma-Aldrich Chemie GmbH, Buchs, Switzerland) and incubated at 37 °C for an additional hour to eliminate extracellular bacteria. Then, the cells were washed twice with PBS, lysed by adding 500 μL 0.1% (v/v) Triton X-100 per well and incubated at rt for 5 min (Boudeau et al., 2003; Gagnon et al., 2013). After incubation, samples were collected, diluted and seeded on TSA plates for bacterial colony counts. The colonies were counted and normalized to the number of eukaryotic cells present in the well.

### Statistical analysis

Each experiment was performed at least in triplicate. Statistical significance was determined using the One-Way ANOVA test on GraphPad Prism 9 statistical software package (GraphPad Software, Inc., San Diego, CA, USA). To assess the statistical significance of adhesion and invasion assays, the Mann-Withney test was used. Data were expressed as means ± Standard Deviation (SD). Multiple comparisons of group mean differences were analyzed by one-way analysis of variance (ANOVA), and Dunnett’s multiple comparisons test was used to compare paired samples. Differences were considered significant when the p-value was less than 0.05.

## Results

### MR335 exerts antiviral activity against different respiratory viruses

In the first set of experiments, based on preliminary results and on literature (Martina et al., 2021, Martina et al., 2022, Martina et al., 2024), we selected MR335 as the reference molecule to be studied in our models of respiratory viruses/bacterial coinfection.

Firstly, we evaluated the cytotoxicity of the molecule on the A549 lung cell line by MTT assay, at different concentrations (500, 400, 300, 200, 150, 100, 80, 60, 40, 20 μM). After 24 h of treatment, cell growth was evaluated and compared with that of untreated cells. Cell viability significantly decreased at MR335 concentrations ≥ 100 μM (p<0.001) (**Figure 1A**). The cytotoxicity test was also performed treating cells with DMSO at the same volumes used for each MR335 dilution, as solvent control curve. In this case, the results showed significantly reduced cell viability (p<0.005) for the volumes of DMSO used for the MR335 higher concentrations (500 and 400 μM) (**Figure 1B**). Based on these results, the range of concentrations chosen for antiviral activity was 10 to 60 μM, showing no cytotoxicity on A549 cell line (**Figure 1**). Next, the antiviral activity of MR335 was tested against different RNA respiratory viruses, i.e. human and avian influenza A virus strains, the seasonal coronavirus HCoV-229E and parainfluenza virus type 1. Although in the influenza A/PR8/H1N1 virus model of infection, the IC_50_ for MR335 was 17.3 µM (**Supplementary Table S1**), we chose to use for all the viruses tested in the study, the concentration of 40 μM to obtain the most reduction of viral titer. Permissive cells to respiratory viruses were infected and treated with the compound, as described in the Materials and Methods section. After 24 h, supernatants were collected, and viral replication was evaluated using different assays.

**Figure 1:**
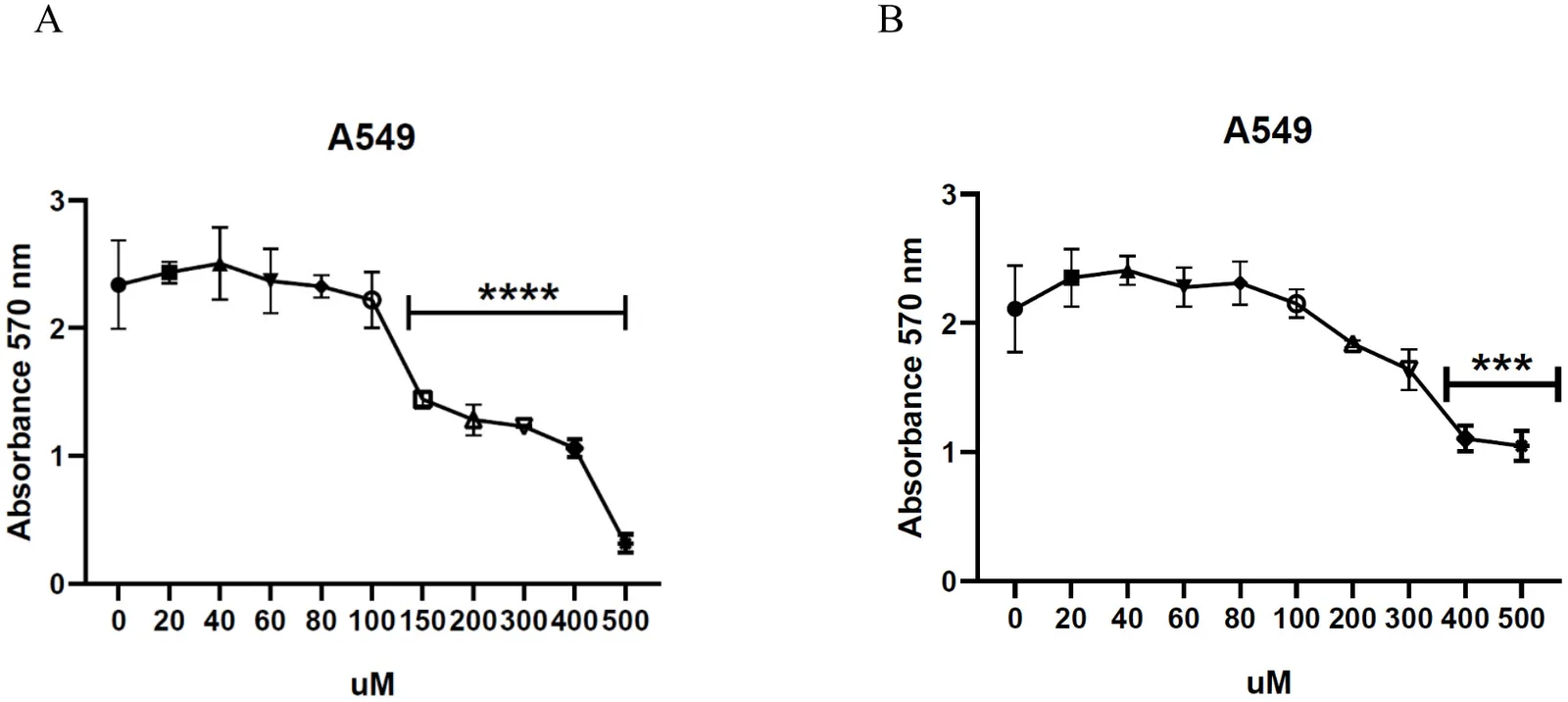
MTT assay on A549 cells treated with MR335 **(A)** or diluent solvent DMSO **(B)** at different concentrations (500, 400, 300, 200, 150, 100, 80, 60, 40, 20 μM).

As shown in **Figure 2A**, MR335 markedly reduced the cytopathic effect induced by influenza A strains infection compared to the untreated controls (indicated as PR8/H1N1 or pH1N1). Consistently, viral titers, measured by HAU assay, in the corresponding supernatants were strongly decreased compared to untreated infected cells (range of reduction in infectivity 75-91.7%) (**Figure 2A**, below images). The TCID_50_ assay also revealed a reduction of viral titer (about 0.5-1.5 log) in infected treated condition (**Figure 2B**).

**Figure 2:**
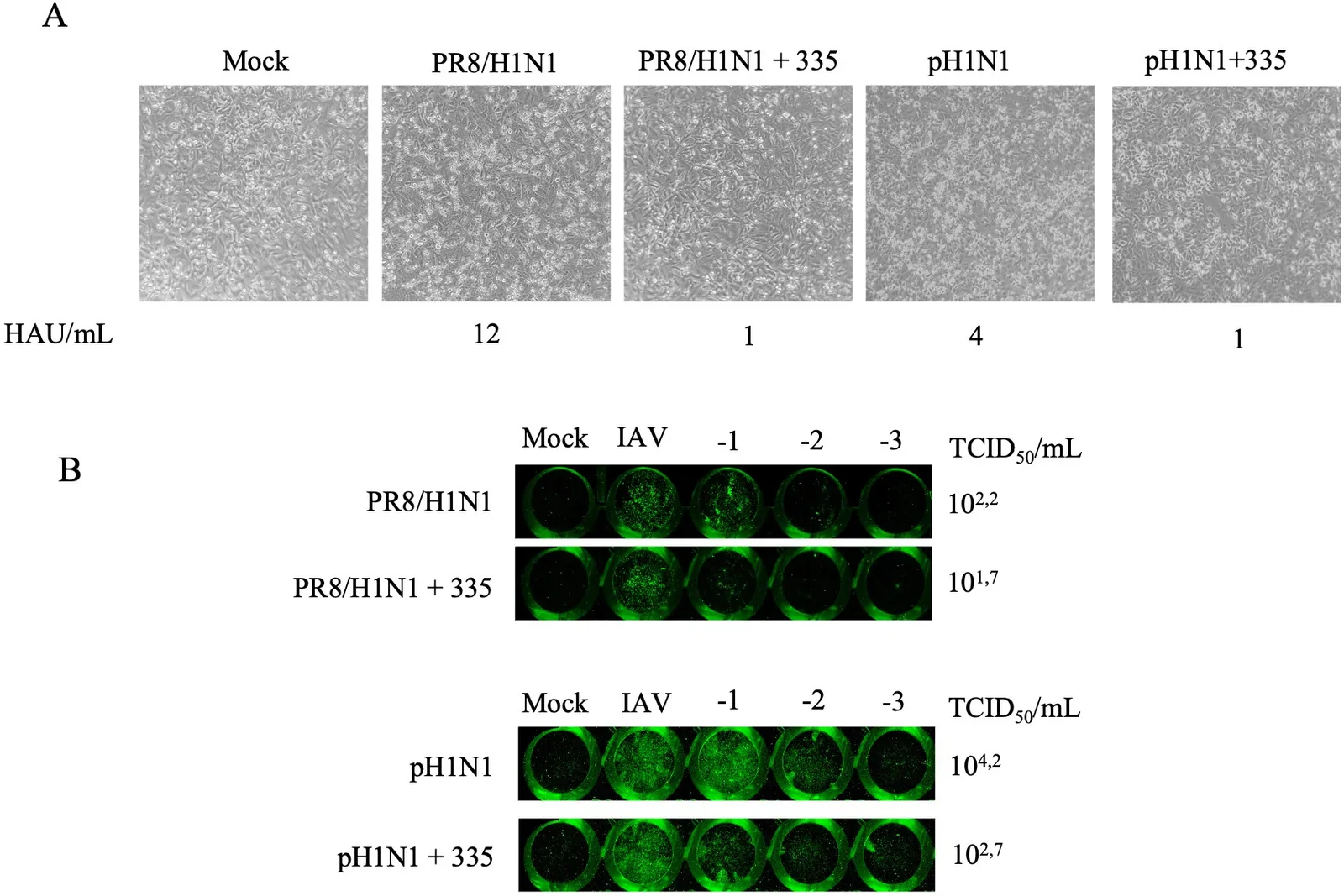
**(A)** Representative light microscopy images acquired using a 10x objective, of PR8/H1N1 or pH1N1 induced cytopathic effect on A549 cells treated or not with MR335 (40 μM) for 24 h post-infection. The relative viral titer measured by HAU assay in the supernatants of A549 infected and treated cells is reported below the images. **(B)** TCID_50_ assay on MDCK cells by using the same supernatants analyzed in **(A)** The same plate was stained with anti-HA antibody and analyzed by Odyssey imaging.

The antiviral activity was also confirmed by using ICW assay in A549 cells that had been infected with either the PR8 or pH1N1 strains. Specifically, the viral infectivity of the supernatants of infected and MR335 treated A549 cells, were used to infect a fresh new cell monolayer. The expression of viral protein (hemagglutinin; HA) was quantified as a percentage of relative fluorescence units (RFU) analyzed by ICW, as described in the material and methods section. As shown in **Figure 3A**, with respect to untreated conditions (considered as 100%), the percentage of HA RFU was reduced by 35% and 30% in PR8 or pH1N1 treated conditions, respectively. Consistent with these findings, RT-qPCR analysis revealed a significant reduction in HA mRNA expression in MR335-treated PR8- or pH1N1-infected cells (**Figure 3B**).

**Figure 3:**
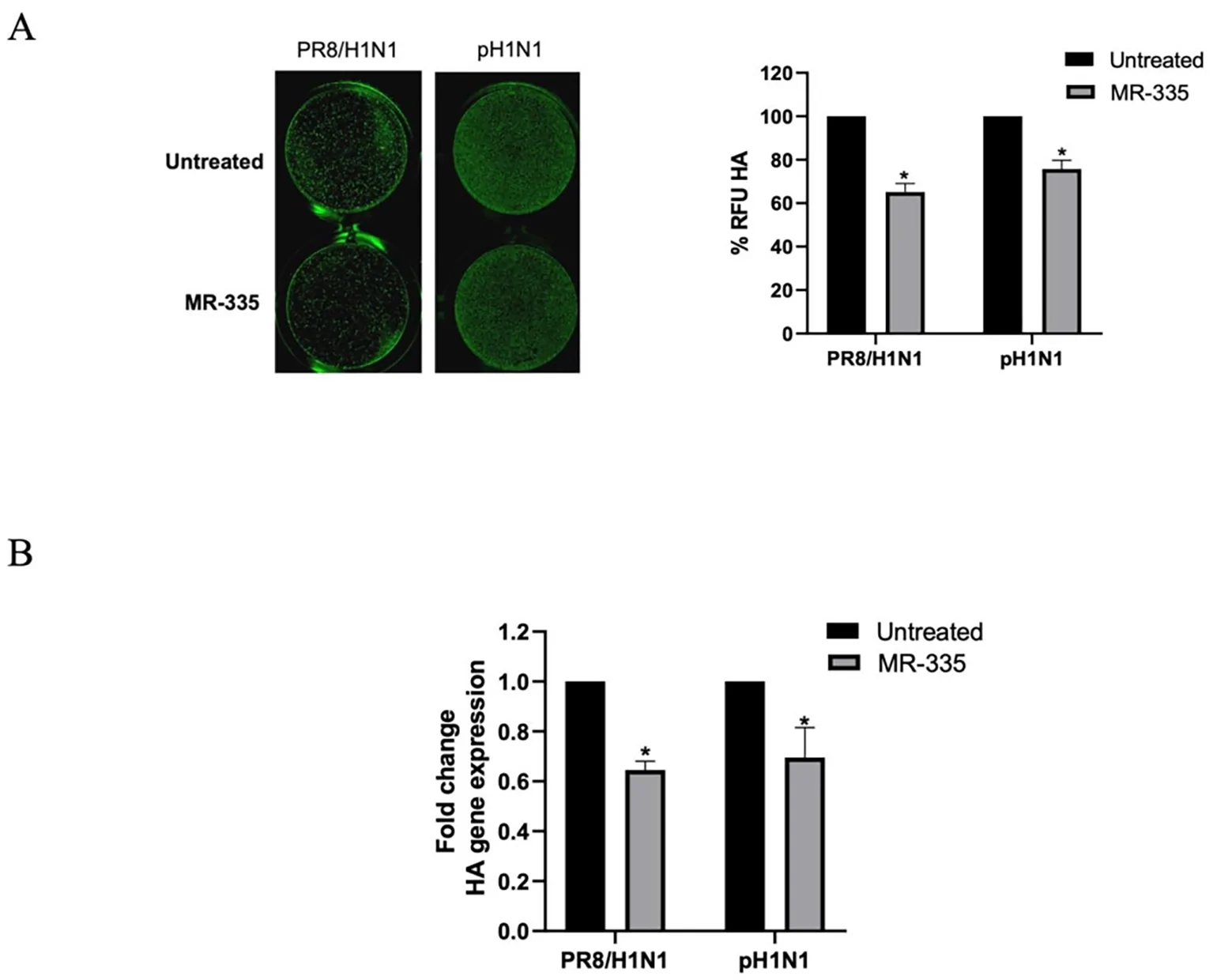
**(A)** In Cell Western (ICW) assay on A549 cells infected with supernatants of PR8/H1N1 or pH1N1 treated or not with MR335 (40 μM) for 24 h. The expression of viral proteins was analyzed by ICW, using LI- COR Image Studio Software. The percentage of relative fluorescence units (RFU) was calculated in comparison to untreated-infected cells (considered 100%). **(B)** Analysis of HA mRNA expression by RT-qPCR in infected (PR8 or pH1N1 virus infections) untreated or treated conditions. Data are expressed as mean ± S.D, obtained from two independent experiments (*p < 0.05 vs. untreated).

To further confirm the MR335 broad spectrum antiviral activity, the compound was evaluated in other respiratory virus infection models. Firstly, MR335 compound was tested in a seasonal coronavirus infection model. Specifically, BEAS-2B cells were infected with HCoV-229E and after 1 h of viral adsorption MR335 (40 μM) was added for 24 h. As reported in **Figure 4A**, western blot analysis of viral proteins Spike and Nucleoprotein (N), showed a drastic decrease in their expression levels in cells treated with MR335 compared to untreated and solvent control treatment.

**Figure 4:**
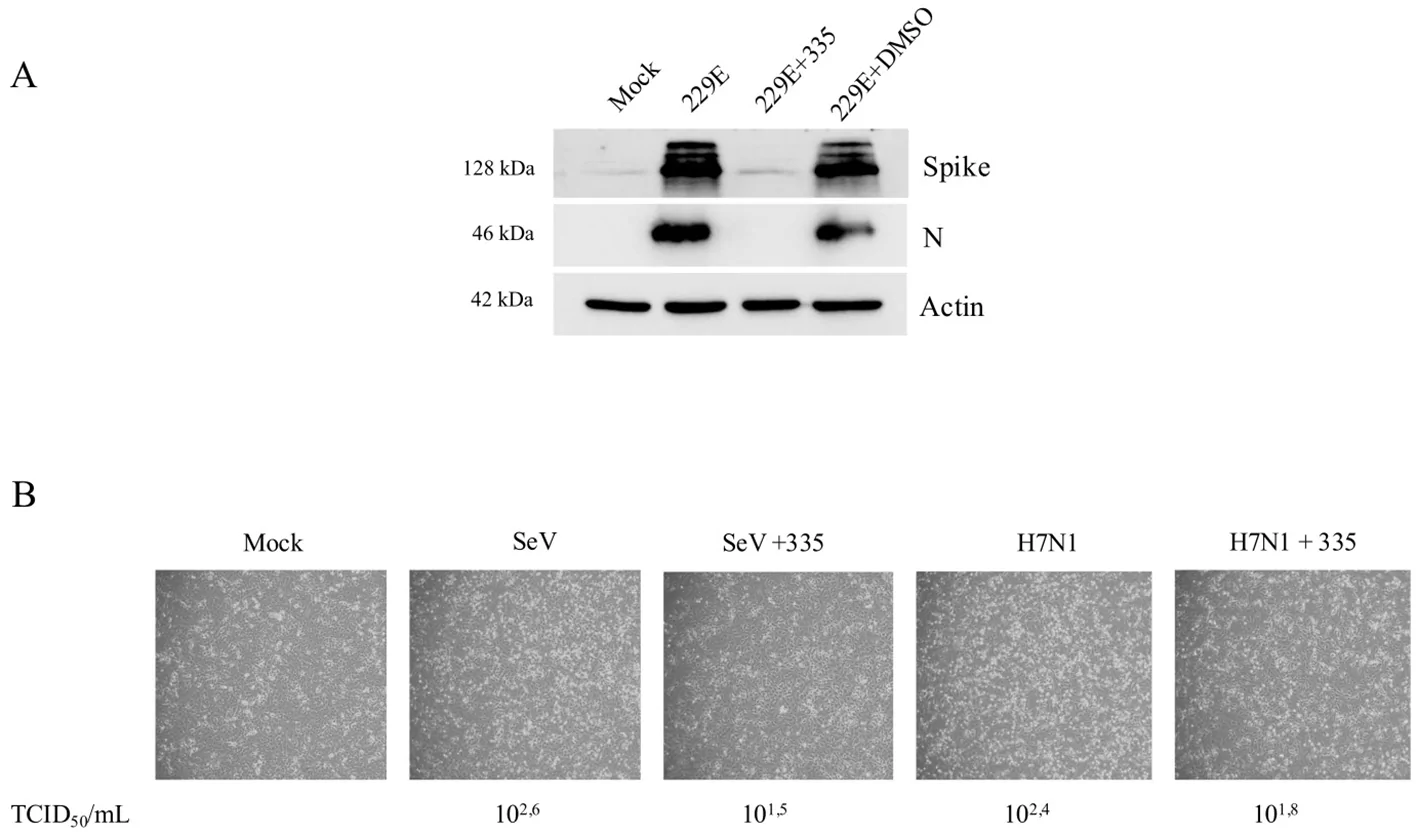
**(A)** Western blot of Spike and Nucleoprotein (N) on BEAS-2B cells infected with HCoV-229E and treated for 24 h with MR 335 (40 μM) compound. **(B)** Representative images acquired using a 10x objective, of SeV or H7N1 induced cytopathic effect on A549 cells treated or not with MR335 (40 μM) for 24 h post-infection. The relative viral titer measured by TCID_50_ assay in the supernatants of A549 infected and treated cells is reported below the respective images.

Then, a reduction of cytopathic effect following MR335 treatment was also observed during infection with avian influenza A/parrot/Ulster/73 (H7N1) and with parainfluenza virus Sendai type 1 (SeV) (**Figure 4B**). Accordingly, the TCID50 assay (below the images) showed a reduction of 1.1 log and 0.6 log in cells infected with SeV or H7N1, respectively.

Based on the results obtained from different viral infection models, MR335 confirmed a broad-spectrum activity against several RNA viruses and was considered useful for further studies on bacterial infection and co-infection models.

### MR335 shows antibacterial activity against *S. aureus* at high concentrations

Next, MR335 was investigated as an antibacterial agent against *S. aureus*. Therefore, the compound was put in contact at different concentrations with *S. aureus* strain for 24 h, as described in Methods. As shown in **Figure 5**, a significant reduction of bacterial load by 5 orders of magnitude (from 10^9^ CFU/ml to 10^4^ CFU/ml), compared with untreated control growth (p<0.01), was found only at concentrations between 400 and 500 μM. The other concentrations tested (300, 200, 150, 100 μM) also showed a strong reduction in bacterial growth (from 10^9^ CFU/ml to 10^7^ CFU/ml) compared to the control (p<0.01), although to a lesser extent than the 400 and 500 μM concentrations.

**Figure 5:**
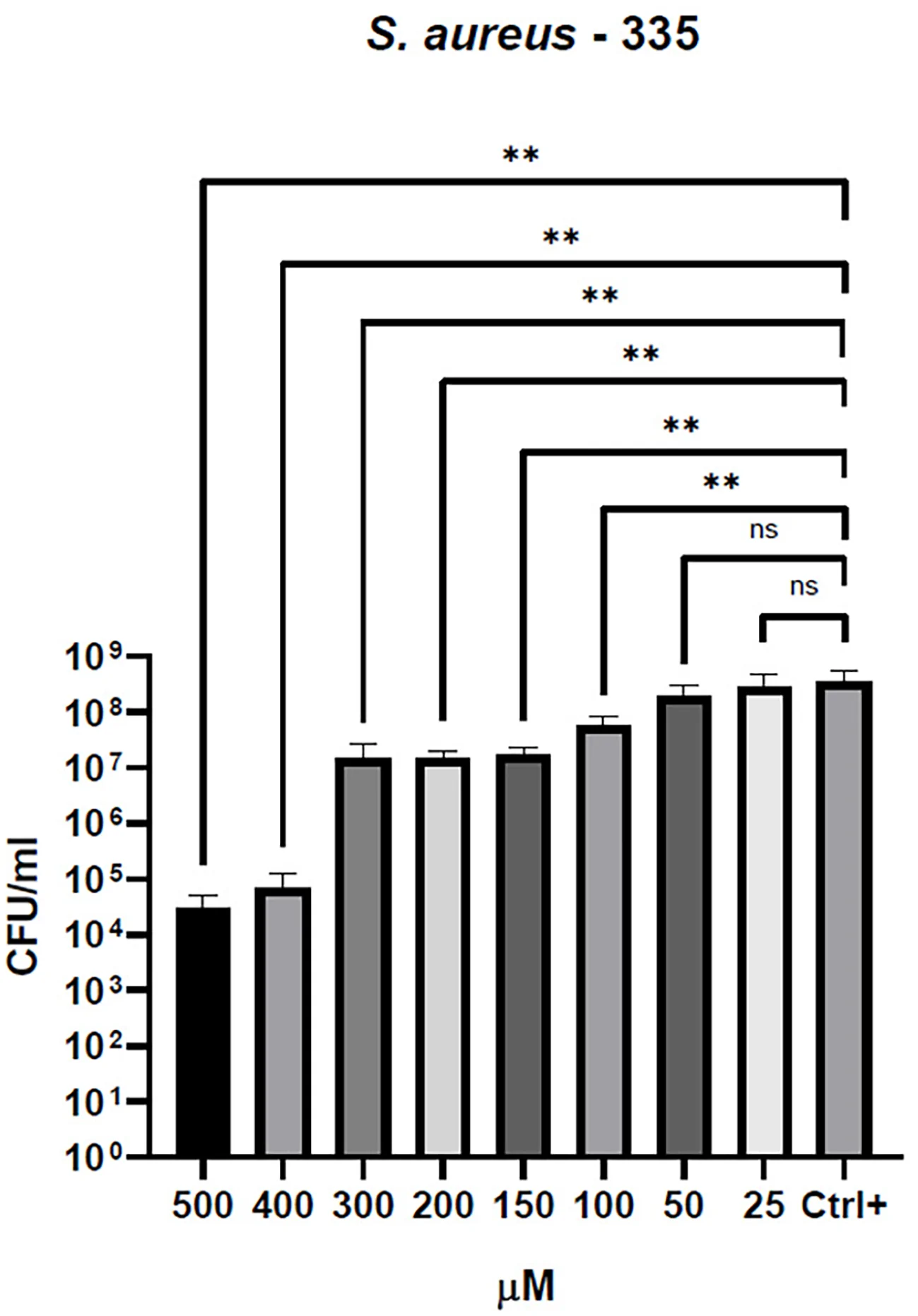
*S. aureus* bacterial growth (CFU/mL) *versus* different concentrations of the bithiazole compound MR335 (micromolar) **p<0.01. Ctrl+: positive control of the bacterial growth, culture not treated with MR335.

Interestingly, the lowest concentrations tested (50 and 25 μM), that had been effective against respiratory virus replication, did not show a significant reduction in *S. aureus* bacterial growth.

Indeed, the antibacterial activity of MR335 against *S. aureus* ATCC 6538 was evidenced at concentrations > 100 μM and, from this concentration, its activity increased in a dose-dependent manner.

### MR335 treatment inhibits the biofilm of *S. aureus*

Based on the results of the antibacterial activity of MR335 obtained in planktonic assays, three intermediate concentrations (500, 300 and 50 μM) were used to verify its activity on *S. aureus* biofilm. Prevention activity of biofilm formation was observed at MR335 concentrations of 500 (**Figure 6A**) and 300 μM (**Figure 6B**). Prevention was only observed when MR335 was added after the first 4 h of biofilm formation (A, After) or when it was present before and after (BA, Before and After) the first 4 h of biofilm formation (p<0.001). When bithiazole was added during the first phase of biofilm formation (the first 4 h) and then removed (B, Before), no significant decrease in biomass was observed. At the concentration of 50 μM, MR335 showed no activity in preventing biofilm formation under any of the three conditions analyzed (**Figure 6C**).

**Figure 6:**
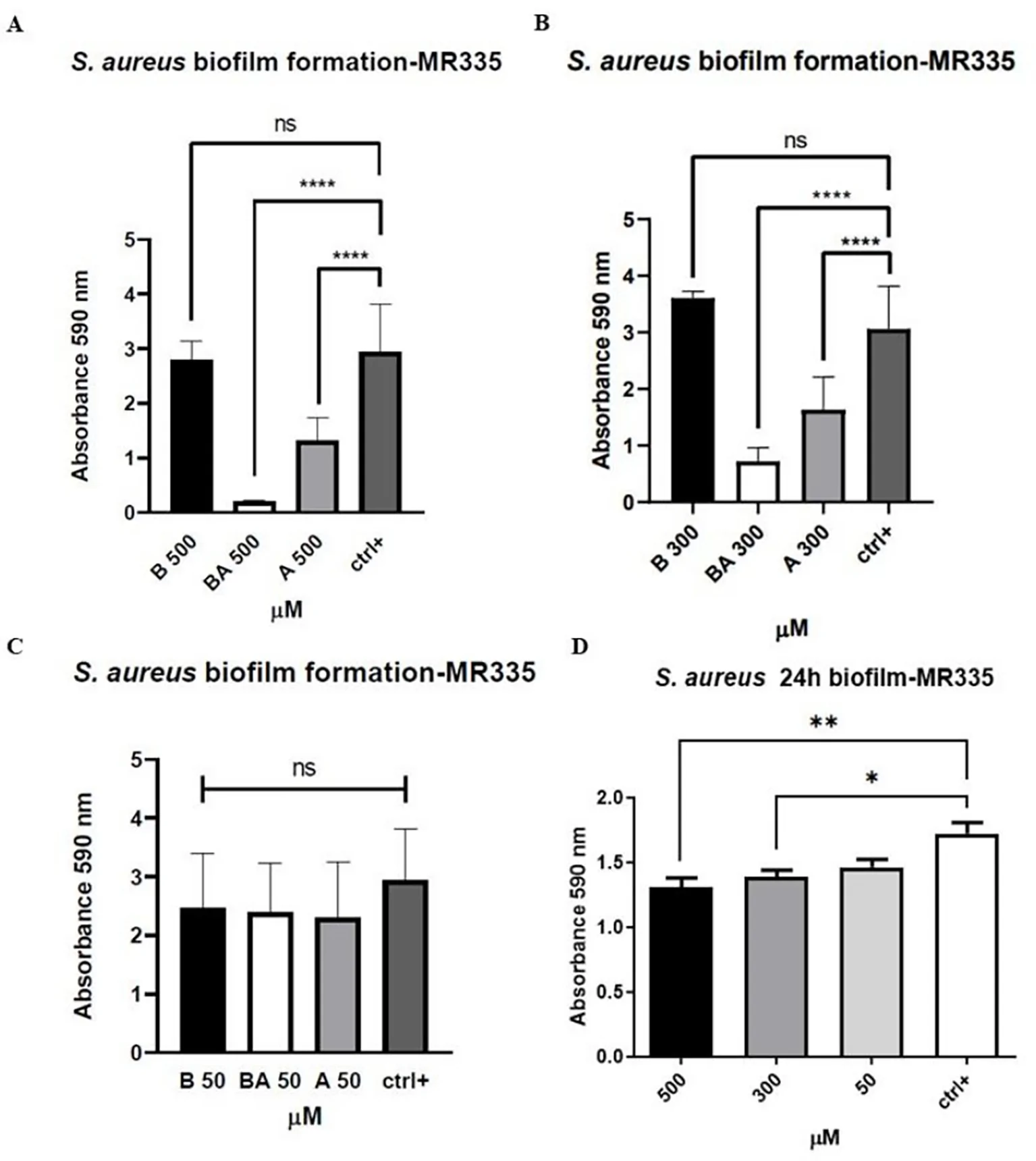
Activity of MR335 in preventing biofilm formation at concentrations of 500 **(A)**, 300 **(B)** and 50 μM **(C)**. **(D)** Activity of MR335 at concentrations of 500, 300 and 50 μM on *S. aureus* biofilm preformed for 24h.

Regarding the antibacterial activity of MR335 on *S. aureus* preformed biofilms, the compound was significantly effective at highest concentrations (500 and 300 μM), as shown by the decrease in biofilm biomass (**Figure 6D**), but with a lower activity respect to biofilm prevention assays (**Figures 6A, B**).

All the results indicated that MR335 exhibits antibacterial activity only when added at high concentrations, evidencing a major activity in the disruption of a mature biofilm rather than in the prevention of initial bacterial attachment.

### MR335 activity on respiratory virus - *S. aureus* coinfection models

Once established the antimicrobial activity of MR335 for viral and bacterial pathogens, coinfection models of respiratory viruses (specifically influenza virus or seasonal coronavirus) and *S. aureus* were set up to investigate the efficacy of the compound to counteract the synergistic interactions between the pathogens. For this purpose, as previously described A549 or BEAS-2B cells were infected with PR8/H1N1 virus or HCoV-229E together with compound MR335 at a concentration of 40 μM, not cytotoxic for the cells and effective against viral replication but not against bacterial growth. After 22 h of viral infection, the cell monolayers were infected with *S. aureus*. At the end of the 2 h of bacterial superinfection, supernatants were collected for viral infectivity analysis and bacterial adhesion and invasion were evaluated. Concurrently, the infection was also conducted in the presence of *S. aureus* alone or the virus alone, to evaluate the influence of the compound in the absence of co-infection.

As shown in **Figure 7**, the adhesion and invasion assays demonstrated a statistically significant increase in the adhesive and invasive capacity of *S. aureus* in the presence of both viruses, PR8 (**Figure 7A**) or HCoV-229E (**Figure 7B**). Results seem to indicate a synergistic activity between pathogens during the co-infection, resulting in an increase of *S. aureus* virulence.

**Figure 7:**
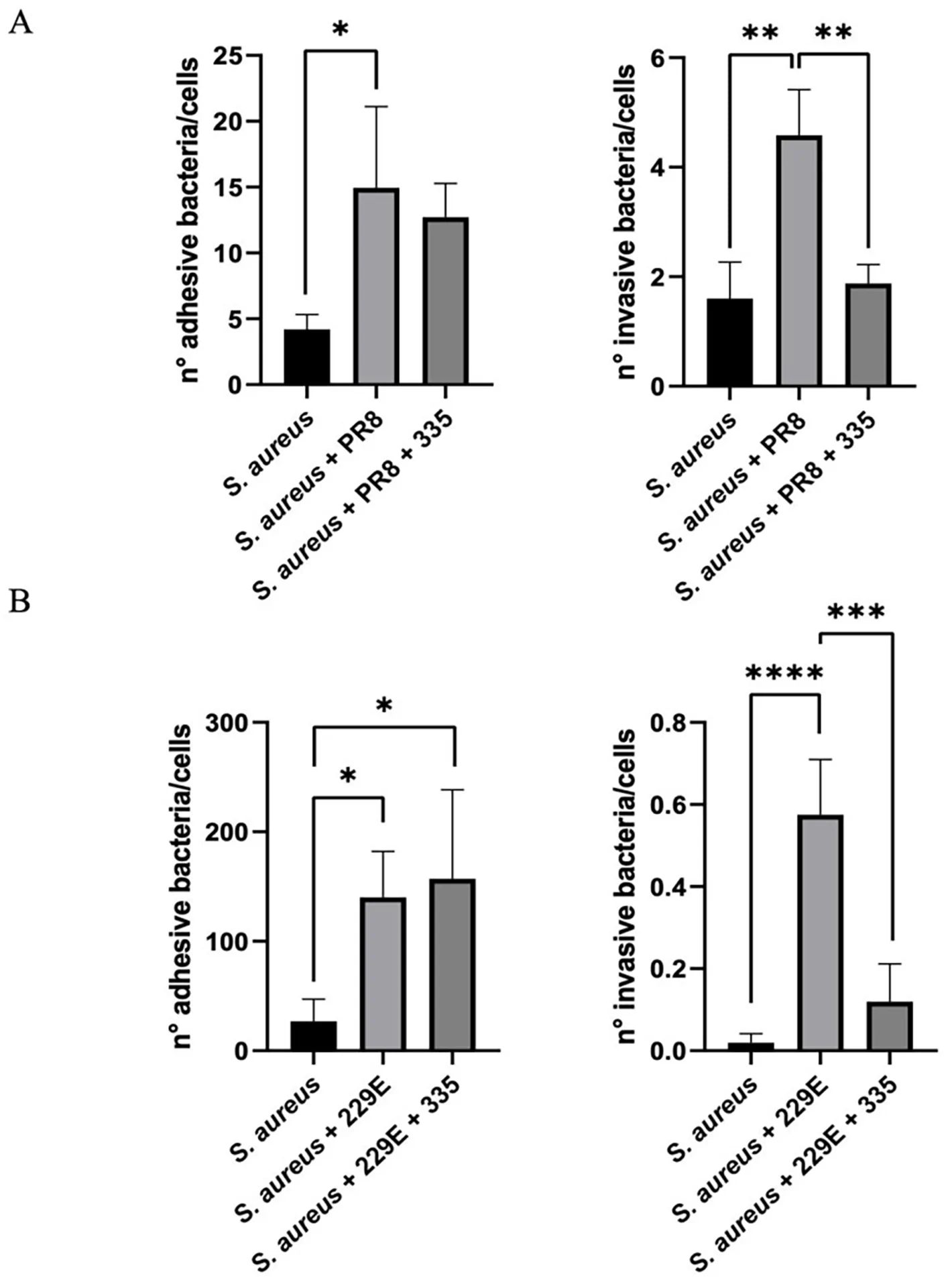
Number of *S. aureus* bacteria able to adhere (left) or invade (right) per cell in *S. aureus* PR8 **(A)** or *S. aureus* 229E **(B)** co-infection models (*p<0.05 and **p<0.01 vs *S. aureus*).

When the compound was added during the co-infection, while the level of *S. aureus* adhesion to infected cells was not affected by MR335-treatment (40 µM) (**Figures 7A, B**, left graphs), the invasion level of *S. aureus* was significantly decreased by MR335-treatment (p < 0.01) (**Figures 7A, B**, right graphs), suggesting that the bithiazole compound might impair bacterial invasion by interfering with virus/bacteria synergism. In particular, in influenza virus-infected cells treated with MR335 (40 μM), the expression level of hemagglutinin (HA) or total influenza virus proteins (revealed with anti-Flu) was reduced by 34 and 22% respectively. Similar reductions were observed under co-infection conditions (**Figure 8A**). Consistently, in HCoV-229E/*S. aureus* coinfection model: MR335 treatment markedly reduced Spike protein expression in *S. aureus* coinfected cells compared with untreated controls (**Figure 8B**). Furthermore, the gene expression of coronavirus nucleoprotein was significantly reduced in MR335-treated cells infected with HCoV-229E alone or coinfected with *S. aureus*. (**p<0.01; **Figure 8C**). Overall, these results suggest that the bithiazole compound, by acting directly on viral protein expression, might interfere with bacterial superinfection.

**Figure 8:**
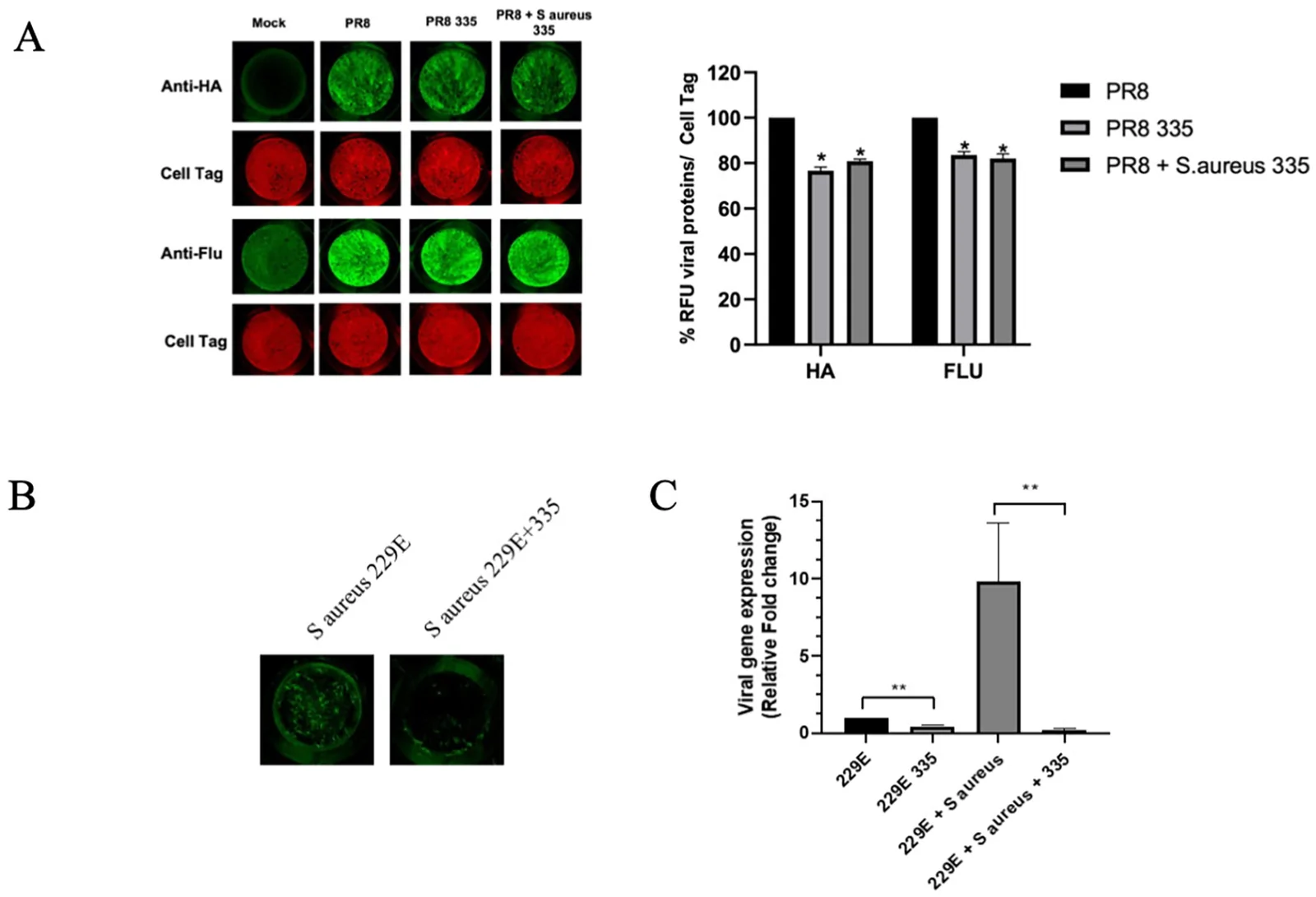
**(A)** In Cell Western assay on A549 cells infected with PR8/H1N1 and *S. aureus* strain and treated for 24 h with MR335 (40 µM) (*p<0.05 vs 229E). Percentage of Hemagglutinin (HA) or anti-influenza (Flu) fluorescence (RFU) is reported in the graph on the right (untreated conditions are reported as 100%). **(B)** In Cell Western assay on BEAS-2B cells infected with 229ECoV strain and treated for 24 h with MR335 (*p<0.05 vs 229E. **(C)** Real-time PCR on cell lysates in co-infection conditions treated or not with MR 335 compound. Viral gene expression of Nucleoprotein was normalized to actin gene used as housekeeping (*p<0.05 and ***p<0.001 and ****p<0.0001 *vs. S. aureus*).

## Discussion

Viral respiratory tract infections are often accompanied by secondary bacterial infection leading to severe pneumonia and an increased risk of shock and mortality (Kuiken and Taubenberger, 2008). Studies investigating the severity of post-influenza bacterial pneumonia have mostly been retrospective and conducted after influenza pandemics (MacIntyre et al., 2018), but *in vitro* investigations on viruses-bacteria co-infections or the evaluation of potential compounds able to interfere with their synergistic interactions are limited. The aim of this study was to establish a model of co-infection (influenza A virus/coronavirus and *S. aureus*) useful to evaluate the potential antiviral and antibacterial ability of the bithiazole derivative (MR335) in counteracting the interaction between both pathogens. A significantly increased adhesive and invasive ability of *S. aureus* was observed when A549 or BEAS-2B cells were pre-infected with influenza A virus or coronavirus, denoting a synergistic interaction between both viruses and *S. aureus*. The increased bacterial invasiveness was markedly stronger in the coronavirus co-infection model (14-fold increase *versus* a 2.3-fold increase in the influenza virus co-infection model). Although different cell lines were used in the two co-infection models, which may have influenced the results, the observed differences between the two viruses could be due to distinct underlying mechanisms related to their different replication strategies, which are currently under investigation.

The increased invasive capacity of *S. aureus* in the co-infection model with influenza A virus has been previously reported by our group and others (Passariello et al., 2006, Passariello et al., 2011, Aleandri et al., 2015; Mulcahy and McLoughlin, 2016). In this study, other than confirming the synergistic interaction between influenza virus and *S. aureus*, we demonstrate, for the first time, the synergistic cooperation between HCoV-229E and *S. aureus*, impacting *S. aureus* pathogenicity. These findings are intriguing, since coronaviruses, particularly the seasonal ones, by spreading via coughing and sneezing, cause mild upper respiratory tract diseases in adults, but sometimes especially in young or aged people or in immuno-compromised individuals, they may cause life-threatening bronchiolitis and pneumonia (Liu et al., 2021). Furthermore, rare cases of severe HCoV-229E infection have been associated with bacterial co-coinfections, including those by carbapenem-resistant *Klebsiella* and MRSA (Sun et al., 2021).

Considering the importance of identifying alternative strategies to the antibiotics currently used to prevent/treat viral/bacterial co-infections, we chose to test the bithiazole compound MR335 in our co-infection models, because of its well-known antimicrobial activity (Martina et al., 2022, Martina et al., 2024). Initially, we evaluated MR335 antiviral and antibacterial activity independently. The compound inhibited viral replication of different RNA viruses, including human and avian influenza strains, parainfluenza virus and coronavirus. Accordingly, viral titers reached their lowest levels at a concentration of 40 μM for both influenza virus and coronavirus. On the contrary, antibacterial activity against *S. aureus* was observed at higher concentrations of the MR335 (≥ 100 μM) and increased in a dose-dependent manner from this concentration onward. Moreover, MR335 at concentrations of 500 and 300 μM was able to inhibit both preformed and forming *S. aureus* biofilm. In the virus-bacterial co-infection models, MR335, at the concentration of 40 μM, was found to negatively interfere with the synergistic relationship between viruses and bacteria, while it did not show any effect on the adhesive/invasive ability of *S. aureus* alone. The observed activity of MR335 in the co-infection models is likely attributable to its antiviral activity. Consequently, by reducing viral protein expression and viral titer, it appears to disrupt the synergistic virus/bacteria relationship, which would result in increased bacterial invasion. Although the mechanism of action underlying MR335 in impairing virus/bacteria synergism remains to be elucidated, we can speculate that certain host cell factors involved in regulating viral replication and favoring bacterial invasion during coinfection, might be modulated by the bithiazole compound. For instance, potential host targets that warrant further investigation include some protein kinases that regulate the traffic of influenza virus nucleoprotein (Nencioni et al., 2009), or play a key role in the creation of replication organelles, which serve as the central hub for the replication of positive-strand RNA viruses, including the common coronaviruses (Han et al., 2024).

In conclusion, although further studies are necessary to better elucidate the mechanisms underlying the virus-bacteria synergy during co-infection, particularly to better explain the stronger effect of coronavirus in promoting *S. aureus* adhesion and invasion, our results provide the first evidence of a synergistic interaction between coronavirus and *S. aureus*. Furthermore, for the first time a bithiazole compound was tested to counteract virus/bacteria co-infections. MR335, when used at a non-cytotoxic concentration that maintains antiviral but not antibacterial activity, was able to impair in our co-infection models the virus/bacterial synergistic interaction by acting specifically on viral replication. Our results suggest that the observed reduction in bacterial invasion seems to be an indirect consequence of its antiviral activity, strengthening the impact of the virus on the pathogenic behavior of bacteria. To enhance the translational significance of our findings, further studies will be necessary, including the use of bacterial clinical isolates such as *S. aureus* strains (MRSA), as well as respiratory viruses circulating in the population. These studies should also be validated in *in vivo* co-infection models.

## Data Availability

The raw data supporting the conclusions of this article will be made available by the authors, without undue reservation.
